# 

**Published:** 1885-03

**Authors:** 


### CONTENTS.

**ORIGINAL COMMUNICATIONS—**

Conditions of Success in Ovariotomy. By Robert Battey, M. D., Rome, Ga. . . . . . . 1
Catarrhal Fever. By Dr. K. P. Moore, Macon, Ga., . . . . . . 8

**SOCIETY REPORTS—**

Macon Medical Society. . . . . . . 13
Atlanta Medical and Surgical Union. . . . . . . 20

**CLINIC REPORTS—**

Fibro-Cystic Tumors of the Jaw. By W. F. Westmoreland, M. D., Atlanta, Ga. . . . . . . 29
Enucleation of the Eyeball. By A. W. Calhoun, M. D., Atlanta, Ga . . . . . . . 32

**CORRESPONDENCE—**

Hydrochlorate of Cocaine. By T. M. McIntosh, M. D., Thomasville, Ga. . . . . . . 34
Dry Dressing for the Umbilical Cord. By J. G. Hopkins, M. D., Thomasville. Ga. . . . . . . 35
Spray in Ovariotomy. By John Homans, M. D., Boston, . . . . 36

**BOOK REVIEWS—**

A System of Practical Medicine. Edited by Wm. Pepper, M. D., LL. D , and Louis Starr, M. D., Philadelphia, . . . . . . . . 37
Manual of Nervous Diseases and an Introduction to Medical Electricity. By A. B. Arnold, M. D , . . . . . . 43
Young Folks' Physiology. By Albert F. Blaisdell, M. D., . . . . . 46
Modern Medical Therapeutics. Edited by J. F. Edwards, M. D., and D G. Brinton, M. D., . . . . . . 47
Bill Arp's Scrap Book, . . . . . . 48

**EDITORIAL—**

Our Journal Volume Two . . . . . . 50

Our Portrait Gallery . . . . . . 52

The Late Dr. E. S. Gaillard . . . . . . 58

Items . . . . . . 59

Our Advertisers . . . . . . 63

